# An *Escherichia coli* Effector Protein EspF May Induce Host DNA Damage via Interaction With SMC1

**DOI:** 10.3389/fmicb.2021.682064

**Published:** 2021-05-26

**Authors:** Muqing Fu, Song Liang, Jiali Wu, Ying Hua, Hanzong Chen, Zhikai Zhang, Jinyue Liu, Xiaoxia Li, Bao Zhang, Wei Zhao, Chengsong Wan

**Affiliations:** ^1^Department of Microbiology, School of Public Health, Southern Medical University, Guangzhou, China; ^2^Key Laboratory of Tropical Disease Research of Guangdong Province, Guangzhou, China

**Keywords:** EHEC (Enterohaemorrhagic *E. coli*), EspF, DNA damage, SMC1, protein interactions

## Abstract

Enterohemorrhagic *Escherichia coli* (EHEC) O157: H7 is an important foodborne pathogen that causes human diarrhea, hemorrhagic colitis, and hemolytic uremic syndrome. EspF is one of the most important effector proteins injected by the Type III Secretion System. It can target mitochondria and nucleoli, stimulate host cells to produce ROS, and promote host cell apoptosis. However, the mechanism of the host-pathogen interaction leading to host oxidative stress and cell cytotoxic effects such as DNA damage remains to be elucidated. Here, we used Cell Counting Kit-8 (CCK-8) assays and 8-oxo-7,8-dihydro-2′-deoxyguanosine (8-OHdG) ELISA to study cell viability and DNA oxidative damage level after exposure to EspF. Western blot and immunofluorescence were also used to determine the level of the DNA damage target protein p-H2AX and cell morphology changes after EspF infection. Moreover, we verified the toxicity in intestinal epithelial cells mediated by EspF infection *in vivo*. In addition, we screened the host proteins that interact with EspF using CoIP-MS. We found that EspF may more depend on its C-terminus to interact with SMC1, and EspF could activate SMC1 phosphorylation and migrate it to the cytoplasm. In summary, this study revealed that EspF might mediate host cell DNA damage and found a new interaction between EspF and the DNA damage repair protein SMC1. Thus, EspF may mediate DNA damage by regulating the subcellular localization and phosphorylation of SMC1.

## Introduction

Enterohemorrhagic *Escherichia coli* (EHEC) O157:H7 is an important foodborne pathogen. It can cause bloody diarrhea, hemorrhagic colitis, and even life-threatening hemolytic uremic syndrome (Tarr et al., 2005). EHEC infection induces an increase in host reactive oxygen species levels and the activation of inflammatory signals, which both play an important role in mediating hemorrhagic colitis (Dahan et al., 2002; Pacheco and Sperandio, 2012). However, the mechanism of this host-pathogen interaction leading to host oxidative stress and DNA damage remains to be clarified.

EHEC O157: H7 adheres to the brush border of intestinal epithelial cells, and approximately 40 effector proteins are injected into host cells using the Type III Secretion System (Slater et al., 2018). EspF is one of the most important virulence factors for EHEC and enteropathogenic *E. coli* (EPEC) (Holmes et al., 2010). The N-terminus of EHEC-secreted effector protein EspF contains a secretory signal (residues 1–21) that helps EspF secretion to host cells. Both a mitochondrial targeting signal (MTS, residues 1–24) and a nucleolar targeting domain (NTD, residues 21–74) enable EspF to target the mitochondria and nucleolus of host cells. Moreover, an essential role for leucine at position 16 was shown for mitochondrial targeting (Nagai et al., 2005), and mutation of this residue to glutamic acid (L16E) has been a pivotal tool for determining which of EspF’s functions are dependent on mitochondrial targeting. The EspF C-terminus (residues 73–248) is composed of four highly homologous proline-rich repeats sequences, each of which contains an SNX9 protein binding site Src homology 3 (SH3) motif and a neuronal Wiskott-Aldrich syndrome protein (N-WASP) binding domain (Hua et al., 2018a).

Based on the structural characteristics of EHEC EspF, it can target mitochondria, destroy the mitochondrial membrane potential, induce the host to produce ROS (Wang et al., 2017), and mediate cell apoptosis (Zhao et al., 2013). In addition, EPEC EspF can also target the nucleolus to regulate ribosomal protein synthesis (Dean et al., 2010). As EPEC often leads to asymptomatic colonization, *E. coli* depletes host cell DNA mismatch repair (MMR) proteins in colonic cell lines and has been detected in colorectal cancer patients (Maddocks et al., 2009). EPEC EspF is critical for the depletion of MMR proteins, which can then lead to mutations in the *Apc* gene. The mechanism of EPEC modulation of the MMR protein is post-transcriptional and depends upon EspF mitochondrial targeting (Maddocks et al., 2013). In addition, EPEC EspF infection significantly increases spontaneous mutation frequency in host cells, which is a typical feature of colorectal tumors (De’Angelis et al., 2018).

In addition to the MMR pathway, Homologous Recombination (HR) and Non-Homologous End Joining are two important DNA double-strand break (DSB) repair pathways (Liu et al., 2017). Among them, HR repair requires a sister chromatid as a template to reduce errors in DNA and only functions in the S phase and G2 phase of the cell cycle (Stefanski et al., 2019). This repair pathway is mediated by serine/threonine kinase of ataxia telangiectasia-mutated (ATM). DSBs can activate ATM kinase and initiate downstream signaling pathways (Burma et al., 2001). SMC1 is an important target of ATM kinase (Yi et al., 2017). It can be phosphorylated by ATM and collaborates with the MRE11, Rad50, and NBS1 complex to regulate DNA replication fork formation and damage repair (Kitagawa and Kastan, 2005).

Although there is evidence that EspF can sequester host cell MMR proteins and cause host microsatellite instability (Maddocks et al., 2013), whether EHEC EspF causes DNA damage through other DNA damage repair proteins and the pathogenicity of EspF-host protein interaction is unclear. We, therefore, analyzed the ability of EspF to cause cell DNA damage and validated the DNA damage of intestinal epithelial cells in mice. Afterward, we demonstrated that EspF interacted with the DNA damage repair protein SMC1 by co-immunoprecipitation combined with mass spectrometry (CoIP-MS). Using CoIP and confocal microscopy, we further confirmed that EspF preferred to mediate the interaction with SMC1 through its C-terminus. EspF can also phosphorylate SMC1 and transfer it to the cytoplasm, affecting the cell’s ability to repair DNA damage in the nucleus. Our research provides new insights into the pathogenesis of EHEC O157:H7 infection.

## Materials and Methods

### Cell Lines and Strains

293T, Caco2 cells, and HT-29 cells were kindly gifted by Professor Bao Zhang, School of Public Health, Southern Medical University, Guangzhou. We cultured 293T and Caco2 cells overnight in DMEM (GIBCO, Waltham, MA, United States) containing 10% fetal bovine serum (FBS) and 1% penicillin and streptomycin at 5% CO_2_ and 37°C. HT-29 cells were cultured overnight in RPMI-1640 (GIBCO) medium containing 10% FBS and 1% penicillin and streptomycin at 5% CO_2_ and 37°C. The pEGFP-N1 plasmid, strain EHEC O157:H7 EDL933, Δ*espF*, and Δ*espF*/*pespF* were stored in our laboratory (Wang et al., 2017). DH5α-competent cells and LA high-fidelity enzyme were purchased from TaKaRa (Shiga, Japan). The restriction enzymes *Eco*RI and *Bam*HI were purchased from Thermo Fisher (Waltham, MA, United States). The primers used in this study were synthesized by Sangon Biotech (Shanghai, China). Gene sequencing was performed by IGE Biotechnology (Guangzhou, China).

### Construction of pEGFP-EspF-3Flag, pEGFP-EspF-N-Flag, and pEGFP-EspF-C-Flag Plasmids

We amplified the *espF* gene, its N-terminus (1 − 219 bp), and C-terminus (220 − 747 bp) using the LA high-fidelity enzyme in the genome of EDL933. Each constructed plasmid was labeled with Flag. Primers *espF-F*: CCGGAATTCGCCACCATGCTTA ATGGAATTAGTAACGCTG and *espF-R*: CGCGGATCCCC GCTACCGCCGCTTCCCTTGTCCTTATCGTCGTCATCCTTG TAATCCTTATCGTCGTCATCCTTGTAATCCTTATCGTCGTC ATCCTTGTAATCCCCTTTCTTCGATTGCTCATA amplified the *espF* gene; primers *espF*/*N-F*: CCGGAATTCGCCACCATG CTTAATGGAATTAGACAC and *espF*/*N-R*: CGCGGATCCCC ACCAGAGCCACCCTTATCGTCGTCATCCTTGTAATCGGGA GTAAATGAAGTCACCTG amplified the *espF*-*N*; and primers *espF*/*C-F*: CCGGAATTCGCCACCATGTCTCGTCCGGCACCG CGCGCCCCCACC and *espF*/*C-R*: CGCGGATCCCCACCTC CCC amplified the *espF*-*C*. The PCR products were digested with *Eco*RI/*Bam*HI and T-linked to the pEGFP-N1 plasmid to generate pEGFP-EspF-3Flag, pEGFP-EspF-N-Flag, and pEGFP-EspF-C-Flag plasmids. Sequencing verified the integrity of the constructed plasmids.

We cultured 293T cells according to standard methods, and the cells were plated on 10 cm^2^ culture dishes (NEST, Hong Kong, China). In accordance with the manufacturer’s instructions, the cells were transfected with pEGFP-EspF-3Flag, pEGFP-EspF-N-Flag, and pEGFP-EspF-C-Flag plasmids using Lipofectamine 3000 (Thermo Fisher).

### Immunofluorescence Assay

Caco2 cells were plated on a confocal dish (35 mm; NEST, Hong Kong) and transfected with pEGFP, pEGFP-EspF, pEGFP-EspF-N, or pEGFP-EspF-C vectors. After 48 h, the cells were gently washed with PBS three times and fixed in 4% paraformaldehyde for 15 min at room temperature, and then blocked with 0.1% Triton X-100 for 30 min. The fixed cells were stained with primary antibody overnight at 4°C (anti-p-H2AX/anti-CK18 [CST, Danvers, MA, United States]; anti-SMC1/anti-p-SMC1 [(Abcam, Cambridge, United Kingdom)], followed by incubation with a secondary antibody for 30 min. Then, the cells were stained with DAPI for 5 min. Cellular co-localization was observed with an FV1000 confocal microscope (Olympus, Tokyo, Japan).

### Western Blot

The protein samples were loaded and separated by sodium dodecyl sulfate-polyacrylamide gel electrophoresis (SDS-PAGE). The electrophoresed proteins were transferred to polyvinylidene fluoride (PVDF) membranes (Merck Millipore, Darmstadt, Germany), then blocked with 5% bovine serum albumin (BSA, Gbcbio, China). Membranes were probed with anti-p-H2AX (CST, United States), anti-H2AX (CST, United States), anti-p-SMC1 (Abcam, United Kingdom), anti-SMC1 (CST, USA), or anti-βactin antibodies (Proteintech, China), followed by a goat anti-rabbit IgG secondary antibody (#SA00001-2, Proteintech, China) or goat anti-mouse IgG secondary antibody (#SA00001-1, Proteintech, China) for 1 h. The blots were visualized using an ECL chemiluminescence substrate kit (#WBKLS0100, Millipore, United States).

### Bacterial Infection

The EHEC O157:H7 EDL933 strain was cultured in LB medium, Δ*espF* and Δ*espF*/*pespF* strains were cultured in LB medium containing kanamycin at 37°C for 12 h, and HT-29/Caco2 cells were seeded in 10-cm^2^ culture dishes. When the cells reached 95% confluence, the cells were infected with bacteria at a multiplicity of infection (MOI) of 100:1 and incubated at 37°C and 5% CO_2_. Then, the medium was aspirated, the cells were gently washed with PBS, and the proteins were collected for immunoblotting.

### Determination of 8-OHdG

The strain EHEC O157:H7 EDL933 was cultured in LB medium, Δ*espF* and Δ*espF*/*pespF* strains were cultured in LB medium containing kanamycin at 37°C for 12 h, and HT-29/Caco2 cells were seeded in 10-cm^2^ culture dishes. When cells reached 95% confluence, the cells were infected with bacteria at an MOI of 100:1 and incubated at 37°C and 5% CO_2_. Celluar 8-oxo-7,8-dihydro-2′-deoxyguanosine (8-OHdG) was determined using a commercial competitive ELISA kit (CUSABIO, Wuhan, China). The concentrations were normalized to a standard sample and expressed in ng/mL.

### Measurement of Cell Viability

We used a Cell Counting Kit-8 (CCK-8) kit (Dojindo, Japan) to measure cell viability. A total of 1,000 cells in a volume of 100 μL per well were cultured in five replicate wells in a 96-well plate in a medium containing 10% FBS. HT-29/Caco2 cells were infected with strains as shown above. Then the cells were incubated in a medium containing gentamicin. The CCK-8 reagent (10 μL) was added to 90 μL DMEM to generate a working solution, of which 100 μL was added per well and incubated for 1.5 h. We performed this assay at 24 h, 48 h, and 72 h after infection.

### Co-immunoprecipitation Assay

Following the instructions of the Pierce CoIP Kit (Thermo Fisher), we prepared 293T cells over-expressing pEGFP-EspF or pEGFP-EspF-N or pEGFP-EspF-C as mentioned above. We added 1 mg of cell lysates to 80 μl of the control agarose resin slurry to remove non-specifically bound proteins. Next, we placed 20 μl of AminoLink resin slurry into the spin column, added 15 μg of immunoglobulin G (IgG) antibody (ABclonal Technology, Woburn, MA, United States)/Flag antibody (Cell Signaling Technology, Danvers, MA, United States), and co-incubated the mixture for 1 h. The protein was added to the spin column and incubated at 4°C overnight. The resin was washed three times with IP lysis and then underwent 10% SDS-PAGE for immunoblotting. Anti-Flag (CST) and anti-SMC1 (CST) antibodies were added, and reactions were incubated overnight at 4°C. After three washes with TBST, the corresponding secondary antibody was added, the mixture was incubated at room temperature for 1 h, and the bands were visualized using an ECL chemical solution.

### Silver Staining

After electrophoresis, SDS-PAGE gels were incubated in a fixing solution for 15 min in a shaker at room temperature. The gel was transferred to a sensitizer using gentle shaking. Then, we added the staining solution and left the mixture for 30 min on a shaker. After staining, the gel was washed with water three times (15 min each) and incubated in a developer solution until bands became visible. The gel was washed in water and then imaged. The different bands were cut out and sent to Huijun Biological Company (Guangzhou, China) for MS.

### Survival Assay of Mice

A total of 30 adult female BALB/c mice (4–5 weeks old, 13.52 ± 0.59g) were randomly divided into three groups (EHEC, △*espF*, and Control group), with 10 mice in every group. The strains were cultured overnight in kanamycin at 37°C. The mice, respectively, received 0.2 mL of the bacterial suspension of different groups (approximately 5 × 10^10^ CFU/mL) by intragastric administration. After 12 h, the mice were infected again with the same amount of bacteria. The Control group received 0.2 mL LB broth. Prior to the gavage, the BALB/c mice were given sterile water containing streptomycin and were injected with mitomycin C (2.5 mg/kg) to enhance susceptibility to bacteria. The survival state of the mice was monitored every 12 h for 7 days. The survival rate and weight change of mice were calculated. After 7 days, the surviving mice were killed and their tissues were taken out. Subsequently, tissue immunohistochemical analysis was performed.

### Statistical Analysis

All statistical analyses were performed using SPSS 20.0 (SPSS Inc., Chicago, IL, USA). The data are expressed as the mean ± SD. Comparisons between groups were made by one-way analysis of variance (ANOVA) followed by Dunnett’s test for separate comparisons. *P* < 0.05 was considered statistically significant.

## Results

### EspF Can Induce Extreme Multi-Nucleation and DNA Damage

**FIGURE 1:**
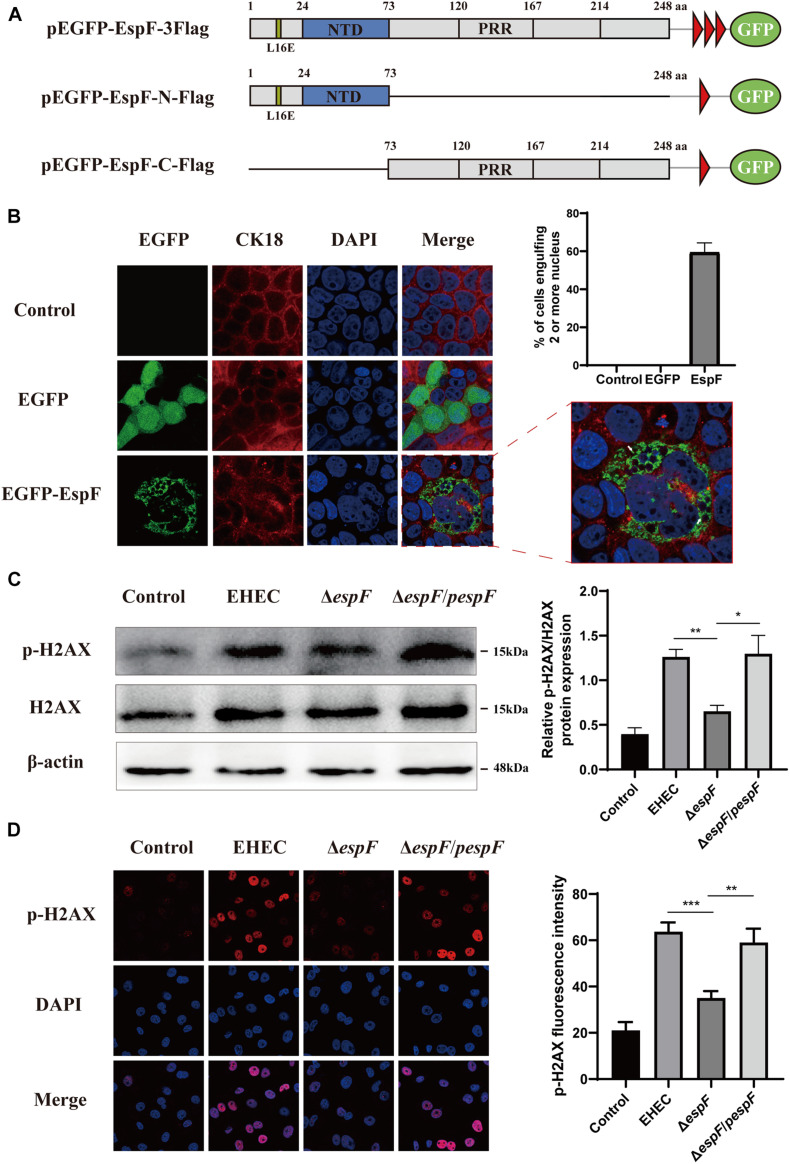
EspF can increase H2AX phosphorylation and mediate cell multi-nucleation. **(A)** Diagram of pEGFP-EspF, pEGFP-EspF-N, and pEGFP-EspF-C plasmids used in this study. pEGFP-EspF, pEGFP-EspF-N, and pEGFP-EspF-C were tagged with Flag. aa: Amino acid. L16E: Mutation of Leucine at position 16 in mitochondrial targeting to Glutamic acid. NTD: Nucleolar Targeting Domain. PRR: Proline-rich repeats. **(B)** Immunofluorescent microscopy of CK18 protein. Caco2 cells transfected with plasmids for 48 h. The nucleus was labeled with DAPI in blue. CK18 was labeled with an anti-CK18 antibody in red. Green indicates fluorescence expressed by the fluorescent plasmid. Images were acquired on an FV1000 confocal laser scanning microscope using a 63 X oil objective. The white arrows represented cell internalization. The grayscale analysis of cells harboring four or more nuclei in cells transfection with EspF as indicated. **(C)** The levels and grayscale analysis of p-H2AX in Caco2 cells infected with strains as indicated. Relative protein levels were quantified using ImageJ software. Data are shown as the mean ± SD of three independent repeats. *P < 0.05, **P < 0.01, ***P < 0.001. **(D)** The fluorescence intensity and grayscale analysis of p-H2AX in Caco2 cells infected with strains for 6 h as indicated. Immunofluorescence was observed with an anti-p-H2AX antibody followed by a secondary antibody (red). Images were acquired on an FV1000 confocal laser scanning microscope using a 63 X oil objective.

The morphological changes of cells are often a manifestation of DNA damage. Mammalian-expression plasmids encoding full-length EspF, or the EspF-N and EspF-C terminus were fused to an N-terminus green fluorescent protein (GFP) with a flag tag (pEGFP-EspF-3Flag, pEGFP-EspF-N-Flag, and pEGFP-EspF-C-Flag) (Figure 1A). Caco2 cells were then transfected with these plasmids, and the medium was changed every two days to maintain cell viability. Cytokeratin 18 is a maker of the cell cytoskeleton. Cells expressing EGFP-EspF on day 2 post-transfection remained mono-nuclear. From day 2 to day 4 post-transfection, cells expressing EGFP-EspF exhibited a progressive increase in cell hypertrophy (maximal diameter) and multi-nucleation (defined as ≥ 15 nuclei). We then quantified the rate of the cell harboring four or more nuclei. Quantification results revealed that 59% ± 5.63% of EspF-expressing cells showed the phenomenon of cell multi-nucleation (Figure 1B), whereas non-transfected cells displayed near-zero levels of internalization. CK18 was rearranged in the cytoplasm and was accompanied by internalized nuclei. In addition, the CK18 protein co-localized with EspF in the cytoplasm. Meanwhile, in the EGFP and Control groups, cells still appeared to be mono-nuclear, and CK18 was distributed continuously along the cell membrane, with a complete structure and clear boundaries.

p-H2AX is a maker protein for DNA double-strand damage. To determine whether EspF could cause DNA damage, we next evaluated the level of p-H2AX in Caco2 cells by infecting them with different strains. Compared with the Δ*espF* group, the levels of p-H2AX in the EHEC and Δ*espF*/*pespF* groups were significantly increased (Figure 1C). Then, we infected Caco2 cells with different strains and observed the distribution of p-H2AX via immunofluorescence. As shown in Figure 1D, in non-infected Caco2 cells (Control), the p-H2AX fluorescence was lower. In the EHEC group, strong p-H2AX fluorescence was observable in the nucleus. By contrast, the Δ*espF* group had weakened punctate fluorescence in the nucleus. The p-H2AX fluorescence was recovered by complementation with *espF*. We then used ImageJ software version 1.8.0 to quantify the fluorescence intensity of p-H2AX in these images. The signal intensity of p-H2AX in the EHEC groups was almost two times higher than that in the Δ*espF* group. These results indicated that EspF may cause DNA damage and morphological changes such as cell multi-nucleation and internalization, along with the rearrangement of the cytoskeleton.

### EspF Causes DNA Oxidative Damage and Decreases Cell Viability

**FIGURE 2:**
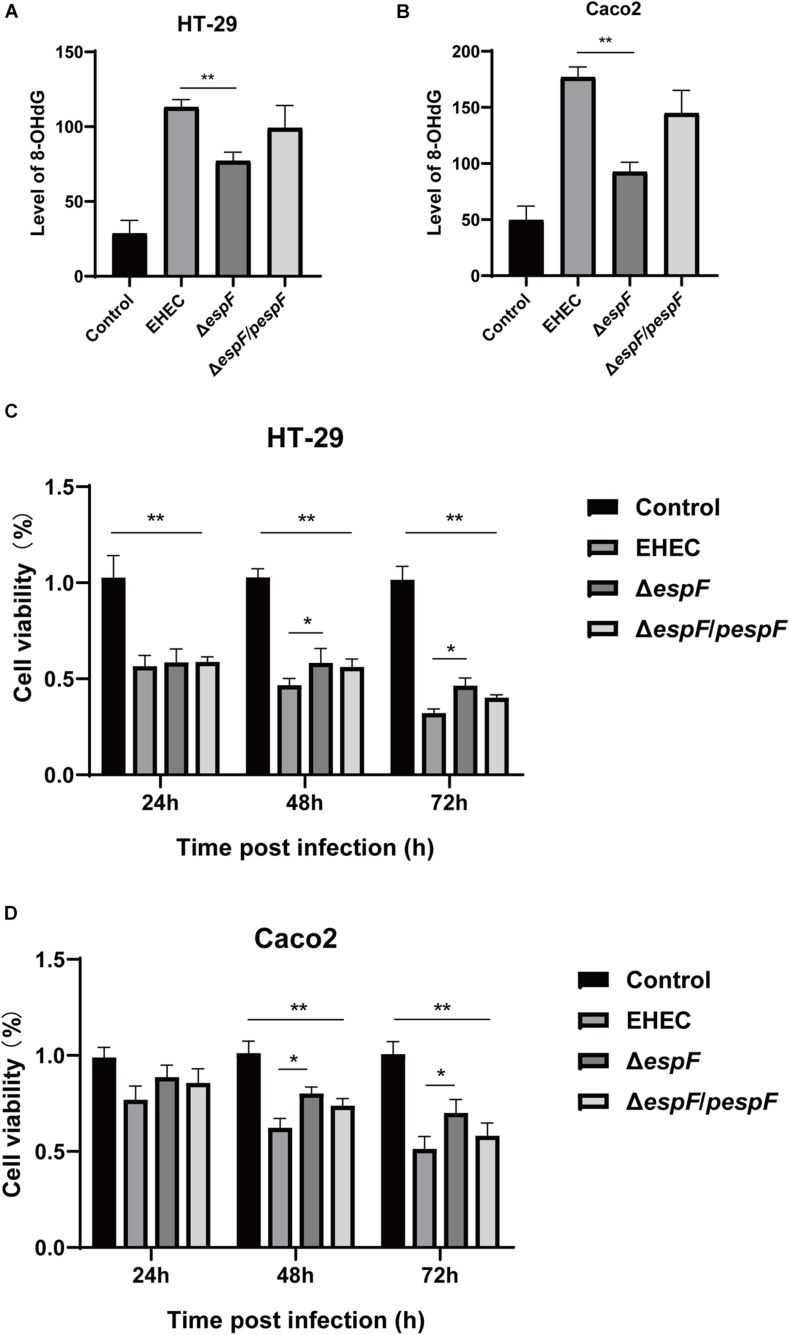
EspF causes DNA oxidative damage and decreases cell viability. **(A)** The level of 8-OHdG in HT-29 cells after infection. HT-29 cells were infected with indicated strains, and DNA was extracted from cells for ELISA. Data are expressed as the mean ± SD from at least three independent experiments. *P < 0.05, **P < 0.01. **(B)** The level of 8-OHdG in Caco2 cells after infection. Caco2 cells were infected with indicated strains for 9 h, and DNA was extracted from cells for ELISA. Data are shown as the mean ± SD of at least three independent experiments. **(C)** Cell viability of HT-29 cells infected with indicated strains. HT-29 cells were infected with strains for 9 h, washed with PBS, and co-incubated in a medium containing gentamicin for 24 h, 48 h, and 72 h. Data are shown as the mean ± SD from at least three independent experiments. **(D)** Cell viability of Caco2 cells infected with strains. Caco2 cells were infected with strains for 9 h as indicated, washed with PBS, and then co-incubated in a medium containing gentamicin for 24 h, 48 h, and 72 h. Data are shown as the mean ± SD from at least three independent experiments.

The most common cell oxidative damage is the formation of mutagenic DNA adducts, including 8-OHdG (Kasai, 1997). We used an 8-OHdG ELISA kit to determine the level of DNA adducts after infection of HT-29 and Caco2 cells, which reflected the level of DNA oxidative damage. In HT-29 cells, the average DNA oxidative damage levels of Control, EHEC, Δ*espF*, and Δ*espF*/*pespF*-infected cells were 28.6, 113.1, 77.0, and 99.1 ng/mL, respectively (Figure 2A). Compared with the EHEC group, the DNA oxidative damage level in the Δ*espF* group was significantly reduced (*P* < 0.01). In Caco2 cells, the average DNA oxidative damage levels of Control, EHEC, Δ*espF*, and Δ*espF*/*pespF*-infected cells were 49.5, 176.8, 92.5, and 144.8 ng/mL, respectively (Figure 2B). The DNA oxidative damage level of the EHEC group was almost two times higher than that of the Δ*espF* group (*P* < 0.05). No differences were observed between the Δ*espF*/*pespF* and Δ*espF* groups, which may be caused by the instability of *espF* gene complementation.

CCK-8 assay can indirectly reflect the number of living cells. The effects of infection with different strains on cell viability were then measured with the CCK-8 assay. After HT-29 cells were infected with these strains, the cells were then treated with gentamicin (50 μg/ml) to remove adherent EHEC, followed by incubation for 24 h, 48 h, or 72 h. After infection with these strains, the cell viability was significantly lower than the Control group (*P* < 0.01). Compared with the EHEC group, the number of cells in Δ*espF* group increased by 2.0%, 11.6%, and 14.2% at 24 h, 48 h, and 72 h, respectively (Figure 2C), and the cell viability at 48 h and 72 h was statistically significant (*P* < 0.05). Similarly, in Caco2 cells, the cell viability of the EHEC group was 11.8%, 18.1%, and 19.5% lower than those of the Δ*espF* group at 24 h, 48 h, and 72 h (*P* < 0.05), respectively (Figure 2D). The Δ*espF*/*pespF* group was slightly lower than Δ*espF*, but the difference was not statistically significant.

### Prediction and Analysis of Protein Interactions With EspF

**FIGURE 3:**
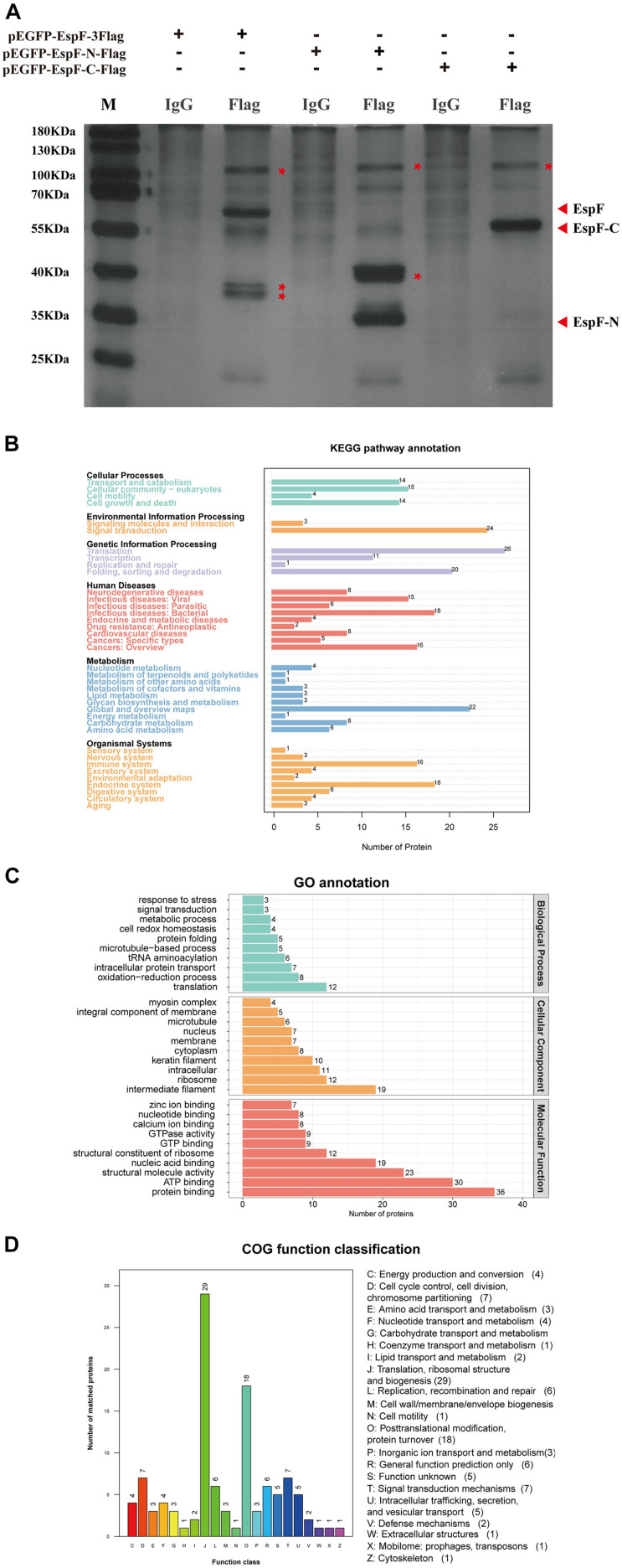
Identification of host proteins that interact with EspF. **(A)** Additional bands from the pEGFP-EspF, pEGFP-EspF-N, and pEGFP-EspF-C groups. The plasmids were transfected into 293T cells. Cell lysates from pEGFP-EspF-transfected cells, pEGFP-EspF-N-transfected cells, or pEGFP-EspF-C-transfected cells were immunoprecipitated with anti-Flag/IgG protein monoclonal antibodies. Immunoprecipitated proteins were isolated by 10% SDS-PAGE and visualized by silver staining. The additional bands were shown with red asterisks (*). **(B)** KEGG analysis of the distribution of interacting protein pathways. **(C)** GO annotation of EspF-interacting proteins in terms of biological processes, cellular components, and molecular functions. **(D)** COG function classification of interacting protein pathways.

After transfecting 293T cells with pEGFP-EspF-3Flag, pEGFP-EspF-N-Flag, and pEGFP-EspF-C-Flag plasmids for 48 h, we lysed cells and precipitated the proteins with Flag and IgG antibodies. These eluted proteins were resolved using 10% SDS-PAGE and visualized by silver staining. Protein expression of pEGFP-EspF-3Flag plasmid was at 60 kDa, that of pEGFP-EspF-N-Flag was at 35 kDa, and that of pEGFP-EspF-C-Flag was at 55 kDa, all of these band sizes were consistent with their anticipated sizes (Figure 3A). Three additional protein bands were specifically precipitated from pEGFP-EspF-transfected cells compared with the IgG group, two additional protein bands were specifically precipitated from pEGFP-EspF-N-transfected cells, and one additional protein band was observed from pEGFP-EspF-C-transfected cells. These additional protein bands in the Flag group and the same position as bands in the IgG group were then cut off and digested by mass spectrometry. The pull-down proteins in the Flag group minus the pull-down proteins in the IgG group were considered to be the putative specific EspF-interacting proteins.

A total of 309 cellular proteins were shown to interact with EspF, while the EspF-N terminus alone interacted with 231 proteins, and 123 proteins interacted with the EspF-C terminus alone (Supplementary Tables 1–3). Kyoto Encyclopedia of Genes and Genomes (KEGG) pathway annotation of the host proteins interacting with EspF revealed that subclasses associated with metabolism, signal transduction, and the immune system were highly enriched (Figure 3B). Gene Ontology (GO) annotation analysis of interacting proteins revealed that subclasses associated with translation (23.1%) and redox process (15.4%) were enriched in Biological Processes (BPs). The most abundant subclasses in Cellular Components (CCs) included intermediate filaments (21.3%) and ribosomes (13.5%). Molecular Functions (MFs) mainly involved protein binding (22.4%) and ATP binding (18.6%) (Figure 3C). Clusters of Orthologous Groups (COG) analysis showed that the interacting proteins were mainly involved in translation, ribosomal structure, and post-translational modification (Figure 3D).

Among these proteins, Structural maintenance of chromosome protein 1A (SMC1) had a high interaction score with EspF. SMC1 is considered to be a chromosomal structural protein (Watrin and Peters, 2006). When cells are stimulated by DNA damage, SMC1 can be phosphorylated by ATM and plays a role in DNA damage repair in the ATM/NBS1 branch of the DSB repair pathway.

### SMC1 Is a Novel EspF-Interacting Protein That May Interact via the EspF-C Terminus

**FIGURE 4:**
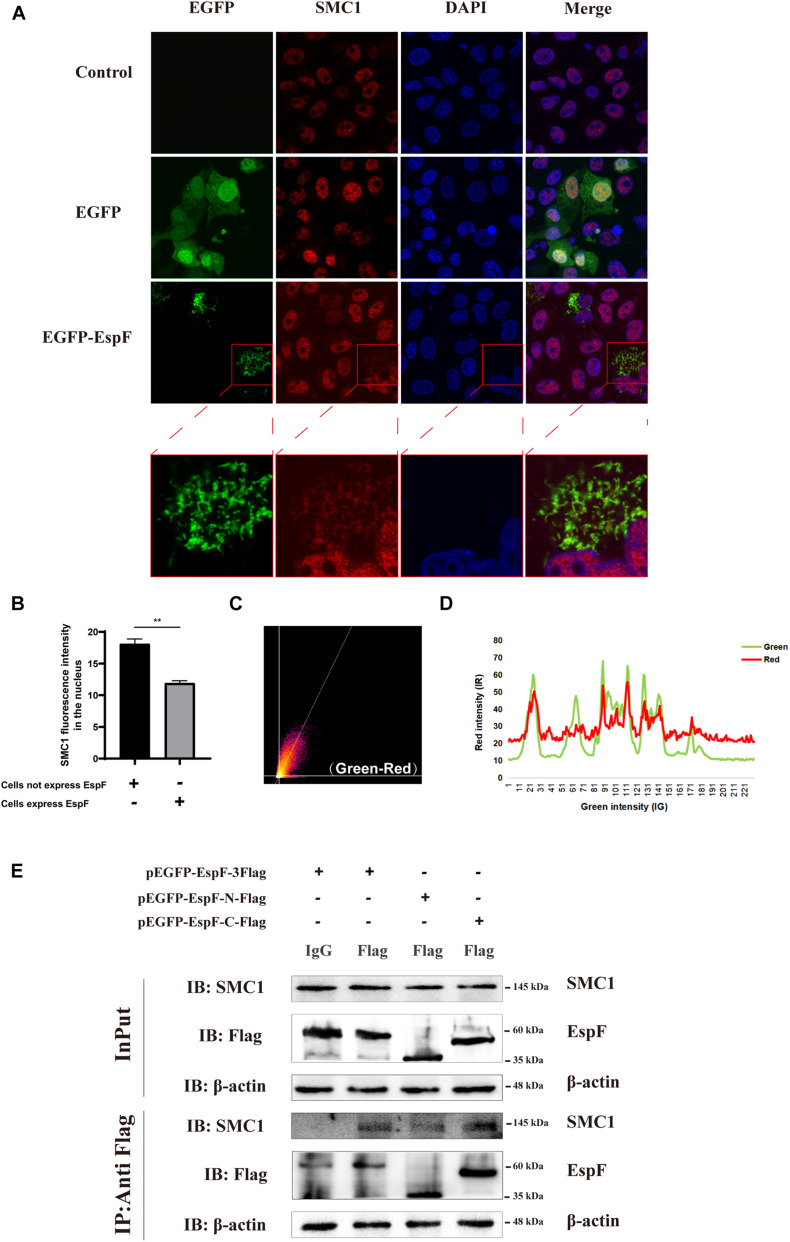
EspF may interact with SMC1 via its C terminus. **(A)** EspF and SMC1 transiently co-localize in the cytoplasm. Caco2 cells were grown to 70% confluence in mock conditions and then transfected with pEGFP-N1 and pEGFP-EspF. After 48 h, cells were fixed, permeabilized, and immunostained with an anti-SMC1 antibody followed by a secondary antibody (red). Images were acquired on an FV1000 confocal laser scanning microscope using a 63X oil objective. The red boxes in panels A were enlarged in the bottom row. **(B)** EspF translocates SMC1 into the cytoplasm. Caco2 cells were transfected with pEGFP-EspF, then stained with an anti-SMC1 antibody. Grayscale analysis of fluorescence intensity of SMC1 in the nucleus as indicated. **P < 0.01. **(C)** The scatter plot of co-localization between EspF and SMC1. the Pearson’s correlation coefficient for the image above thresholds of EspF and SMC1 was 0.7034. **(D)** The plot profile of fluorescence intensity of red/green. **(E)** EspF may interact with SMC1 via its C terminus by CoIP. pEGFP-EspF-3Flag, pEGFP-EspF-N-Flag, or pEGFP-EspF-C-Flag were transfected into 293T cells for 48 h. Cell proteins were extracted and incubated with Flag/IgG agarose beads. Next, input and IP protein lysis were prepared for 10% SDS-PAGE gel. The indicated interacting proteins were immunoprecipitated with indicated antibodies (IB); normal mouse IgG was used as a negative control.

Our MS results revealed that EspF could interact with SMC1. Confocal microscopy analysis and CoIP further validated this interaction between EspF and SMC1. We transfected pEGFP-EspF plasmids into Caco2 cells, and our IF results showed that SMC1 was mostly distributed in the nucleus in the Control group, but after transfection with EspF, SMC1 protein was partially transferred from the nucleus to the cytoplasm, and SMC1 and EspF co-localized in the cytoplasm (Figure 4A). We then quantified the fluorescence intensity of SMC1 protein in the nucleus, and the level of SMC1 in the cells expressing EspF was significantly lower than that in cells not expressing EspF, *P* < 0.01 (Figure 4B).

We next quantified the amount of co-localization between EspF and SMC1 in the cytoplasm. We used ImageJ to make a scatter plot of this co-localization (Figure 4C), and the Pearson’s correlation coefficient (R^2^) for the images above the thresholds for EspF and SMC1 was 0.7034. Moreover, the plot profile of co-localization showed that the red fluorescence intensity (SMC1) fluctuated with the green fluorescence intensity (EspF) (Figure 4D). These quantitative results clearly verified the interaction between EspF and SMC1.

We next used CoIP to confirm the interaction between EspF and SMC1 and the specific interaction domains required for this interaction. We transfected pEGFP-EspF-3Flag, pEGFP-EspF-N-Flag, and pEGFP-EspF-C-Flag into 293T cells and then obtained cell lysates. As shown in Figure 4E, we observed that the EspF and the EspF-C terminus co-precipitated with SMC1, whereas the N-terminus interacted very weakly with SMC1. Thus, SMC1 preferred to interact with the EspF-C terminus. The biological regulation of host SMC1 protein by EspF, however, still needs to be further studied.

### EspF Can Activate SMC1 Phosphorylation and Change Its Subcellular Localization

**FIGURE 5:**
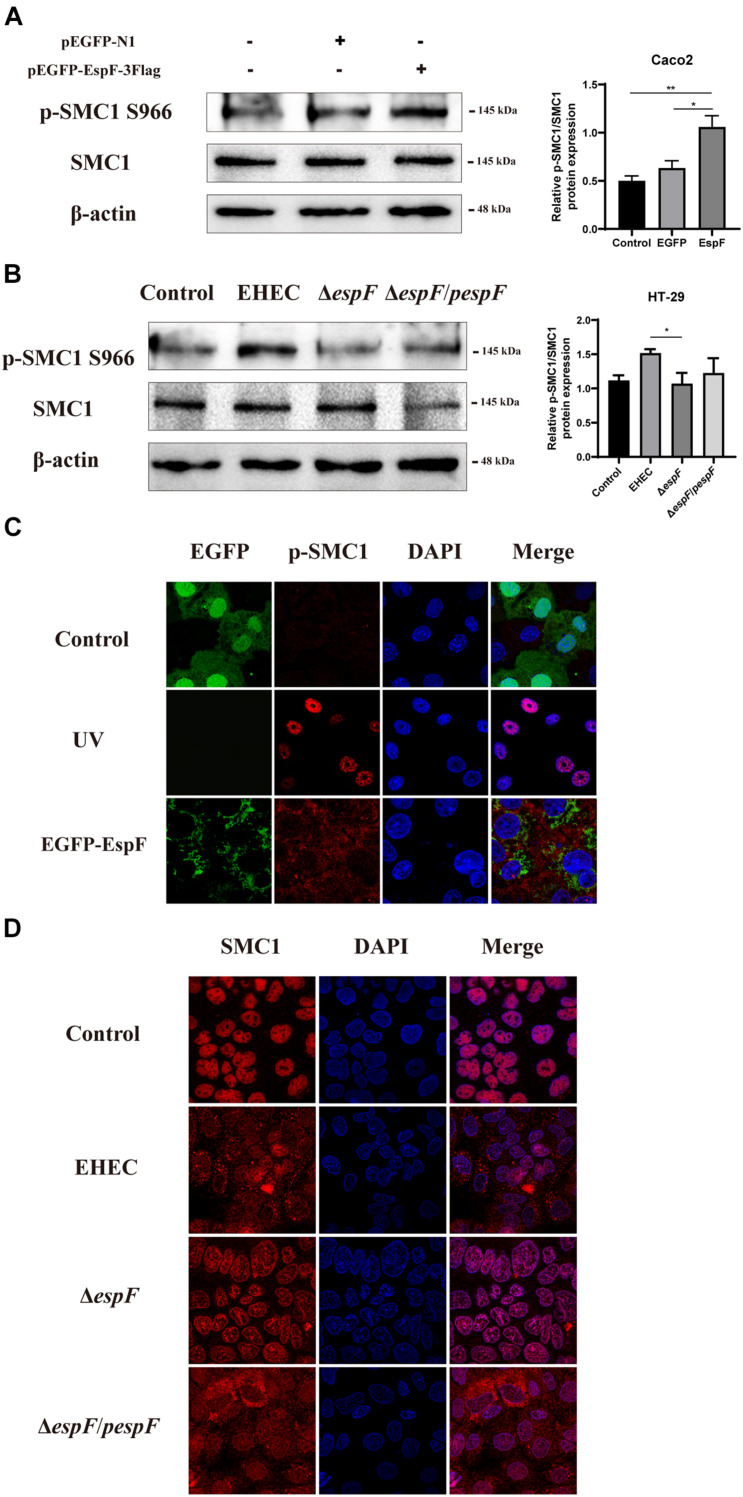
EspF activates the phosphorylation of SMC1 and redistributes it to the cytoplasm. **(A)** EspF increases the phosphorylation of SMC1 in Caco2 cells. pEGFP-N1 and pEGFP-EspF were transfected into Caco2 cells for 48 h. The expression levels and grayscale analysis of p-SMC1/SMC1 were as indicated. Relative protein levels were quantified using ImageJ software. Data are shown as the mean ± SD from three independent repeats. *P < 0.05, **P < 0.01. **(B)** EspF increases the phosphorylation of SMC1 in HT-29 cells. HT-29 cells were infected with indicated strains. The expression levels and grayscale analysis of p-SMC1/SMC1 were as indicated. Relative protein levels were quantified using ImageJ software. Data are shown as the mean ± SD from three independent repeats. *P < 0.05. **(C)** EspF alters the subcellular localization of p-SMC1. Immunofluorescence with anti-p-SMC1 antibody followed with a secondary antibody (red). Images were acquired on an FV1000 confocal laser scanning microscope using a 63X oil objective. **(D)** EspF alters the sub-cellular localization of SMC1. Immunofluorescence using an anti-SMC1 antibody followed by a secondary antibody (red). Images were acquired on an FV1000 confocal laser scanning microscope using a 63X oil objective.

To determine how EspF regulates the level of SMC1 in host cells, we measured SMC1 and its phosphorylation levels after transfecting pEGFP-EspF into Caco2 cells. After transfection with EspF, the level of SMC1 remained unchanged, but the phosphorylation of SMC1 was significantly higher compared with the EGFP group (*P* < 0.05) (Figure 5A). We confirmed this result by infecting HT-29 cells with the strains. The phosphorylation of SMC1 in cells infected with EHEC was higher than in those infected with Δ*espF* cells (*P* < 0.05) (Figure 5B).

We next observed the localization of phosphorylation of SMC1 in Caco2 cells by transfecting with pEGFP-EspF. As shown in Figure 5C, the level of p-SMC1 expression was low in the EGFP group. We used UV treatment as a positive control to induce DSBs. After exposure to 10 J/m^2^ of UV, p-SMC1 was distributed in a punctate manner in the nucleus. While the level of p-SMC1 in the EspF group was significantly increased, p-SMC1 was typically transferred to the cytoplasm and co-localized with EspF protein. The above results verified that EspF could activate SMC1 phosphorylation and transfer it to the cytoplasm.

Then, we infected Caco2 cells with the strains. SMC1 was expressed in the nucleus in the Control and Δ*espF* groups, while in EHEC-infected cells, SMC1 was redistributed more from the nucleus to the cytoplasm. Also, the phenotype of SMC1 in cells infected with Δ*espF*/*pespF* was consistent with EHEC, suggesting that EspF prevented SMC1 from properly playing its DNA damage repair role in the nucleus (Figure 5D).

These findings suggested that EspF could change the subcellular localization of SMC1 protein from the nucleus to the cytoplasm, thereby preventing p-SMC1 from playing its role in the nucleus in DNA damage repair.

### EspF Induces DNA Damage in Intestinal Epithelial Cells *in vivo*

**FIGURE 6:**
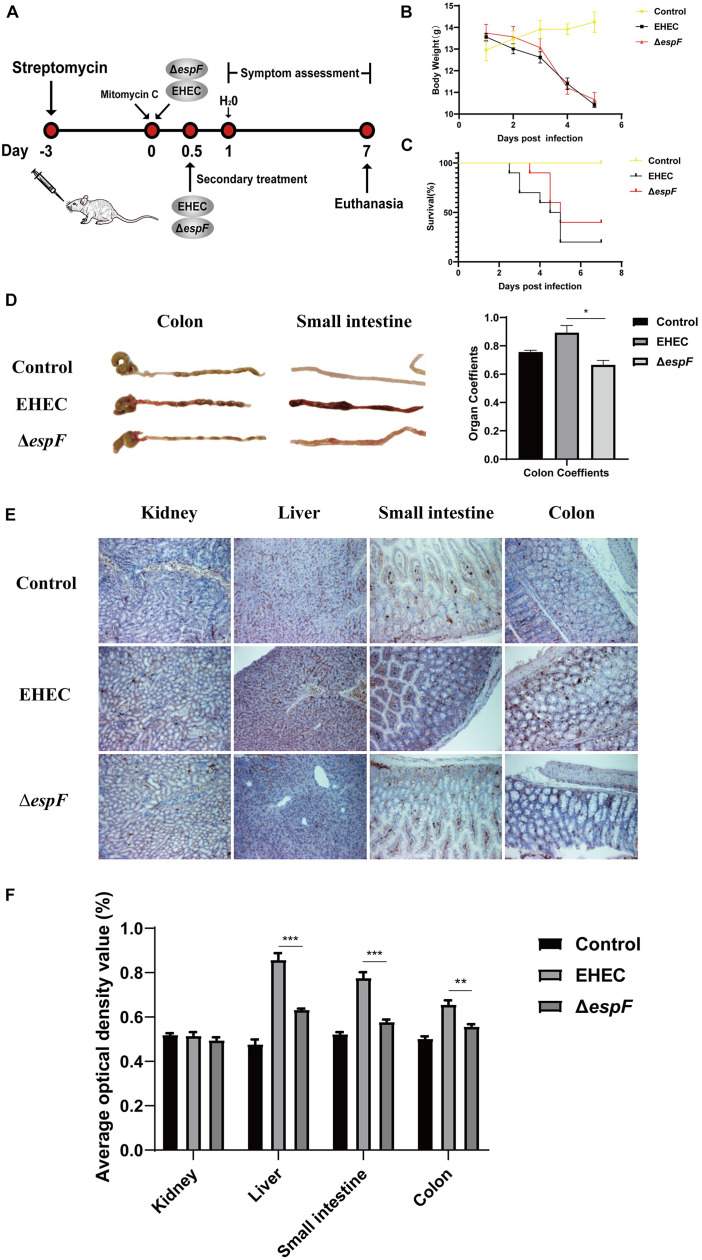
Pathogenicity of EHEC O157:H7 mutant strains in BALB/c mice. **(A)** Construction of an animal model of EHEC O157:H7 hemorrhagic enteritis. **(B)** The bodyweight of the mice at 7 days post-infection. **(C)** Survival curves of BALB/c mice gavage with indicated strains. **(D)** The intestinal appearance and colon coefficient in mice. **(E)** Immunohistochemistry for p-H2AX in different tissues. **(F)** Grayscale analysis of p-H2AX in different tissues. The average optical density value was quantified using ImageJ software. Data are shown as the mean ± SD from three independent repeats. *P < 0.05, **P < 0.01, ***P < 0.001.

To test our *in vitro* findings *in vivo*, we constructed an animal model of EHEC O157:H7 hemorrhagic enteritis based on our previous method (Wang et al., 2017). We improved this protocol by using streptomycin in sterile drinking water before gavage (Figure 6A), making EHEC O157:H7 the dominant intestinal bacteria strain and increasing the susceptibility of the BALB/c mice (Bohnhoff and Miller, 1962).

After the gavage of the strains, the mice showed sparse hair, poor mobility, and hind limb hemiplegia. The weight of mice decreased continually over the seven days. The weight of mice in the EHEC groups decreased faster, while the weight loss of the Δ*espF* group increased rapidly after the fourth day of infection (Figure 6B). Mice in the EHEC group had a rapid onset of disease, and the mice died on the 2.5 d of infection. The onset of the mice in the Δ*espF* group was, however, delayed. The mortality of the mice in the EHEC and Δ*espF* groups were 80 and 60%, respectively (Figure 6C).

After dissecting the mice, we found that the colon of the Control group was a normal yellow, and there was no redness or swelling in the small intestine or colon. In the EHEC group, the colon had edema and became bloody, the stool was black and granular, and small bowel lesions were more obvious, presenting as hemorrhagic colitis. The appearance of the colon in the Δ*espF* group was similar to that in the Control group, the colon lesions were mild, and the small bowel wall only had partial edema (Figure 6D). We weighed the colon of the mice and calculated the organ coefficient based on body weight (Figure 6D). The results showed that the colon coefficients of the EHEC group were significantly higher than those of the Δ*espF* group and Control group (P < 0.05), indicating that EspF could cause thickening of the colonic mucosa in mice and mediate severe edema and inflammation. These results demonstrated that EspF enhanced lethality and induced intestinal lesions in BALB/c mice.

We next examined the kidney, liver, small intestine, and colon tissue for p-H2AX levels using immunohistochemistry. The results showed that the normal lobule of the liver could be seen in the Control group, and only a few brown particles were seen in the nucleus. Compared with the Δ*espF* group, the phosphorylation of H2AX in the EHEC group was significantly increased. The intestinal tissue damage of mice was also more obvious (Figure 6E). We quantified the results of this immunohistochemistry by gray value analysis. Except for the kidney, the phosphorylation of H2AX in the EHEC group was significantly higher than that in the Δ*espF* group (*P* < 0.05) (Figure 6F), suggesting that EspF may mediate different degrees of DNA damage in various tissues.

## Discussion

It has been reported that EspF can lead to an increase in spontaneous mutations in host cells (Maddocks et al., 2013). However, the molecular mechanism of EspF-mediated DNA damage and microsatellite instability remains still unclear.

Cell morphological changes are also one of the manifestations of DNA damage, such as cell multi-nucleation and cell hypertrophy (Horn and Triantafyllopoulou, 2018). Some cells escape from cell cycle arrest and enter abnormal mitosis. This process is characterized by significant changes in cell morphology (Tyrer et al., 2014). Affected cells swell due to inappropriate cytokinesis, changing actin stress fibers, and form micro-nuclei and multi-nuclei. Studies have shown that EPEC EspF mediates cell multi-nucleation and this extreme phenotype depends on the C-terminus region of EspF (Dean and Kenny, 2013). Cell fusion relies on N-WASP, the protein that binds to the EspF-C terminus. In addition, EspF collaborates with CK18 and 14-3-3ζ (Viswanathan et al., 2004), resulting in the alteration of the intermediate-filament network. We hypothesized that EHEC EspF could also cause cell multi-nucleation. We transfected pEGFP-N1 and pEGFP-EspF into Caco2 cells, and cells expressing EspF showed multi-nucleation, accompanied by the rearrangement of the CK18 cytoskeleton, while the cells in Control and EGFP groups still maintained a single nucleus and had an intact cytoskeleton. These results indicated that EHEC EspF could induce host cell multi-nucleation and cytoskeletal rearrangement, and provided a basis for understanding EspF-mediated DNA damage.

One of the earliest events that occur after DNA damage is the phosphorylation of Ser139 on histone H2AX (p-H2AX), which is a known marker of DNA damage (Hong et al., 2016). In this study, we infected Caco2 cells with the strains. Compared with the Δ*espF* group, the phosphorylation of H2AX was higher in the EHEC and Δ*espF*/*pespF* groups. Immunofluorescence further confirmed that the EHEC and Δ*espF*/*pespF* groups had stronger red fluorescence signals. Therefore, we verified that EspF may induce host cell DNA damage.

EspF can increase the level of ROS in the host. It is worth noting that excessive ROS can lead to host DNA damage and form the mutagenic DNA adducts of 8-OHdG (Kasai, 1997; Kryston et al., 2011; Hua et al., 2013). The levels of 8-OHdG in the EHEC groups were significantly higher than those in the Δ*espF* group. Due to the instability of *espF* gene complementation, no statistically significant difference was observed between the Δ*espF*/*pespF* and Δ*espF* groups. Caco2 cells are more prone to DNA oxidative damage, which may be related to the characteristics of cell differentiation, such as intestinal cell differentiation and cell polarity (Gagnon et al., 2013).

During the infection of intestinal epithelial cells, the surface properties of EPEC/EHEC induce exogenous apoptotic pathways (Abul-Milh et al., 2001), whereas TIIISS effectors such as EspF and Map trigger intrinsic apoptotic pathways (Wong et al., 2011). EspF disrupts the mitochondrial membrane potential, leading to the release of cytochrome c and the cleavage of caspases 3 and 9, which eventually mediates cell apoptosis (Nougayrède and Donnenberg, 2004). Our research also confirmed that EspF reduced cell viability. Through CCK-8 assays, the cell viability of EHEC-infected cells was shown to be significantly lower than that of the Δ*espF* group at 48 h and 72 h after infection (*P* < 0.05). These results indicated that EspF could cause DNA oxidative damage, induce host cell apoptosis, and reduce cell viability. However, we noticed that compared with the Control group, the Δ*espF* strain still caused a significant decrease in cell viability. As we know, EHEC hemolysin employs outer membrane vesicles to target the mitochondria and cause endothelial and epithelial apoptosis (Bielaszewska et al., 2013). In addition, the EPEC effector Map imports into mitochondria, causing mitochondrial dysfunction and Ca^2+^ efflux into the host cytoplasm, resulting in the release of epidermal growth factors that stimulate the apoptosis signaling pathway (Ramachandran et al., 2020). Thus, activation of cell apoptosis may be a result of multiple factors.

We speculated that EspF may interact with specific host proteins to mediate DNA damage. Therefore, we used CoIP-MS to identify the interactions between EspF and host proteins. Mass spectrometry identified the SNX9, SNX18, 14-3-3, and ANXA6 proteins which had previously been verified to interact with EspF, confirming the credibility of our mass spectrometry results. 309 host cell proteins interacting with EspF were revealed by CoIP-MS. Interacting proteins were mostly involved in metabolic pathways and ribosomal biological processes. We found that EspF interacted strongly with ribosomal RPL, RPS, and EIF family proteins. Previous proteomics studies have shown that the levels of many ribosomal proteins in intestinal cells decrease after EPEC infection (Hardwidge et al., 2004). In cells expressing EspF, pre-rRNA synthesis is blocked, and EspF-dependent EPEC infection reduces the expression level of the ribosomal protein RPL9 and changes the localization of RPS5 and U8 small nucleolar RNA (snoRNA) (Dean et al., 2010). Our results provided further support for the hypothesis that EspF might play its biological role by regulating ribosomal protein synthesis.

SMC1 had a higher score among our interacting proteins. It has also been shown to interact with EspF and be involved in multiple signal pathways via a bimolecular fluorescence complementation method (Hua et al., 2018b). It is shown that the SMC1 protein is specifically implicated in sister chromatid cohesion (Ball and Yokomori, 2008). The cohesin SMC1 is involved in ATM-dependent responses to DNA damage, and sister chromatid cohesive protein complexes play a key role in the HR-mediated repair of DSBs (Jessberger, 2009). DSBs can activate ATM kinase and initiate downstream signaling pathways. These downstream targets include the checkpoint kinase CHK1, CHK2, and SMC1, which regulate DNA replication forks and damage repair (Carrassa and Damia, 2017). We further verified the interaction between EspF and SMC1 by CoIP and immunofluorescence. Moreover, we demonstrated that the functional domain of interaction was most likely through the C-terminus of EspF. It is known that the binding of EspF to SNX9 and N-WASP proteins is based on the c-terminus SH3 and CRIB (Cdc42/rac interaction binding) motifs (Holmes et al., 2010). The EspF-C terminus, thus, may play a role as a scaffold, linking EspF with other proteins and exerting its biological functions.

We applied immunofluorescence and CoIP to further validate the interaction between EspF protein and SMC1 protein. Moreover, the interaction between SMC1 and the EspF-C terminus was stronger than that with the N terminus. In addition, the nuclear level of SMC1 in the cells expressing EspF was significantly lower than that in cells not expressing EspF, *P*<0.01, thus demonstrating the interaction between EspF and SMC1 can change the subcellular localization of SMC1.

We also examined the effect of sub-localization of SMC1 with strain infection. In HT29 cells, SMC1 concentrated in the nucleus in the Control and Δ*espF* groups, whereas EHEC and Δ*espF*/*pespF* infection lead SMC1 to migrate from the nucleus to the cytoplasm. Moreover, the phosphorylation of SMC1 was less expressed in EGFP groups, while it was punctated distributed in the nucleus in the UV treatment group. Transfection of EspF significantly activated the phosphorylation of SMC1 in the cytoplasm and co-localized with EspF. Therefore, we hypothesized that EspF might interact with SMC1 and transfer it to the cytoplasm, which decreased the SMC1 level in the nucleus, reducing the phosphorylation of SMC1 in the nucleus, and thus down-regulating the ability of DNA damage repair and inhibiting the HR pathway repair. However, the exact mechanism of EspF-SMC1 modulation of this deserves further study.

We also demonstrated that EspF may mediate DNA damage in intestinal epithelial cells *in vivo*. Mice have always been the ideal animal model for studying EHEC infections (Wadolkowski et al., 1990; Mohawk and O’Brien, 2011). In this study, we added streptomycin to the drinking water to increase mice’s susceptibility to pathogenic bacteria (Bohnhoff and Miller, 1962). Among these mice, the EHEC group had the highest mortality rate (80%), and the earliest disease onset. The appearance of the colon in the EHEC group showed hemorrhagic colitis such as edema and vacuoles, and the colon coefficient (0.912) was higher than the Δ*espF* group (0.663), *P* < 0.05. Immunohistochemistry for p-H2AX in different tissues showed that, except for the mice kidney, the phosphorylation of H2AX in EHEC groups was significantly increased than Δ*espF* group (*P* < 0.05). These results indicated that EspF can induce DNA damage in intestinal epithelial cells and mediate animal death.

This study did have some limitations. First, compared with EHEC, the expression of the complementation strains △*espF*/*pespF* was unstable. The *espF* gene was cloned into the pBAD33 plasmid to construct a complementary strain (Hua et al., 2015). Perhaps due to the pBAD expression system, the expression level of EspF was controlled by the regulation of L-arabinose, which caused the instability. Second, some of the host proteins that have been confirmed to interact with EspF were not identified in our MS results, such as N-WASP protein (Garber et al., 2018). This may be due to the size of N-WASP being about 55 kDa, it was not included in the bands we interrogated by MS. In addition, some weak interactions may not be detected. Further experiments to verify the interactions between EspF and host cell proteins would support these findings.

**FIGURE 7:**
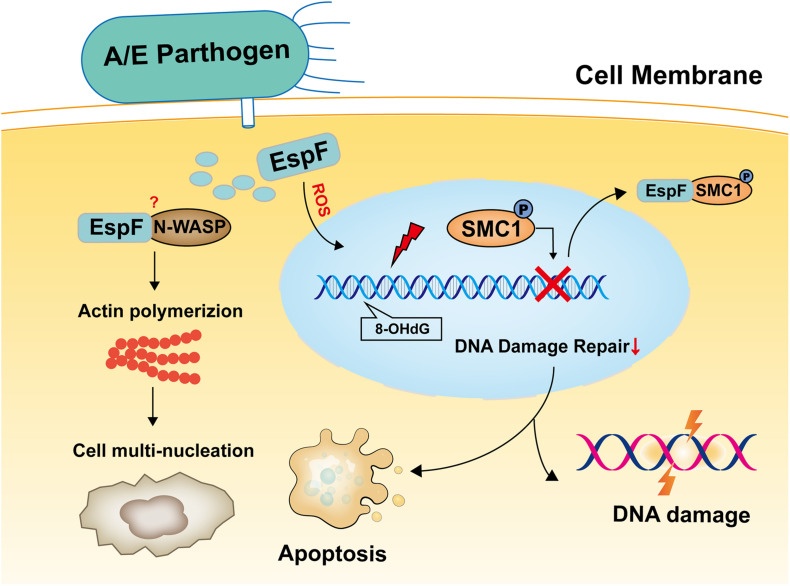
Model of EspF may induce host DNA damage. EspF induces cell multi-nucleation, exacerbates DNA oxidative damage, inhibits cell viability, interacts with SMC1, transfers p-SMC1 into the cytoplasm, then may lead to DNA damage.

In summary, the results reported herein show that EspF induces cell multi-nucleation, exacerbates DNA oxidative damage, inhibits cell viability, and may lead to DNA damage and death in mice (Figure 7). In addition, the host proteins that interact with EspF are identified by CoIP-MS, and we verify that EspF interacts with a novel DNA damage repair protein SMC1. EspF can transfer p-SMC1 to the cytoplasm, making it unable to perform normal DNA damage repair in the nucleus and inhibiting the HR pathway. EspF, therefore, appears to cause DNA damage via interaction with the SMC1 protein, thus contributing to the pathogenicity of EHEC.

## Data Availability Statement

The raw data supporting the conclusions of this article will be made available by the authors, without undue reservation.

## Ethics Statement

The animal study was reviewed and approved by Animal Experimental Committee of Southern Medical University.

## Author Contributions

MF, CW, and YH designed the research and wrote the manuscript. MF, SL, JW, and HC performed the research and conducted the data analysis. ZZ, JL, XL, BZ, and WZ supervised the project and edited the manuscript. All authors contributed to the article and approved the submitted version.

## Conflict of Interest

The authors declare that the research was conducted in the absence of any commercial or financial relationships that could be construed as a potential conflict of interest.

## Supplementary Material

Supplementary Figure 1 Supplementary data for Figure 1. Three independent Western blots to detect p-H2AX and H2AX protein in Caco2 cells.

Supplementary Figure 2 Supplementary data for Figure 4. Three independent CoIP experiments to confirm the interaction between EspF, EspF-N, and the EspF-C terminus and SMC1 protein.

Supplementary Figure 3 Supplementary data for Figure 5. Three independent Western blots to detect p-SMC1 and SMC1 protein in Caco2 cells and HT-29 cells.

Supplementary Figures 4–6 Immunofluorescence figures of internalization phenomenon in Caco2 cells expressing EspF.

Supplementary Figure 7 iTRAQ analysis of the differential proteins of EHEC and Δ*espF* strains infected with HT29 cells in the cell cycle pathway.

Supplementary Table 1 The list of the proteins interacting with EspF protein.

Supplementary Table 2 The list of the proteins interacting with EspF-N protein.

Supplementary Table 3 The list of the proteins interacting with EspF-C protein.
