# Supplementary figures and images for: An Escherichia coli Effector Protein EspF May Induce Host DNA Damage via Interaction With SMC1
Source: Front Microbiol. 2021 May 26;12:682064. doi: 10.3389/fmicb.2021.682064 (PMC8188558; doi:10.3389/fmicb.2021.682064)

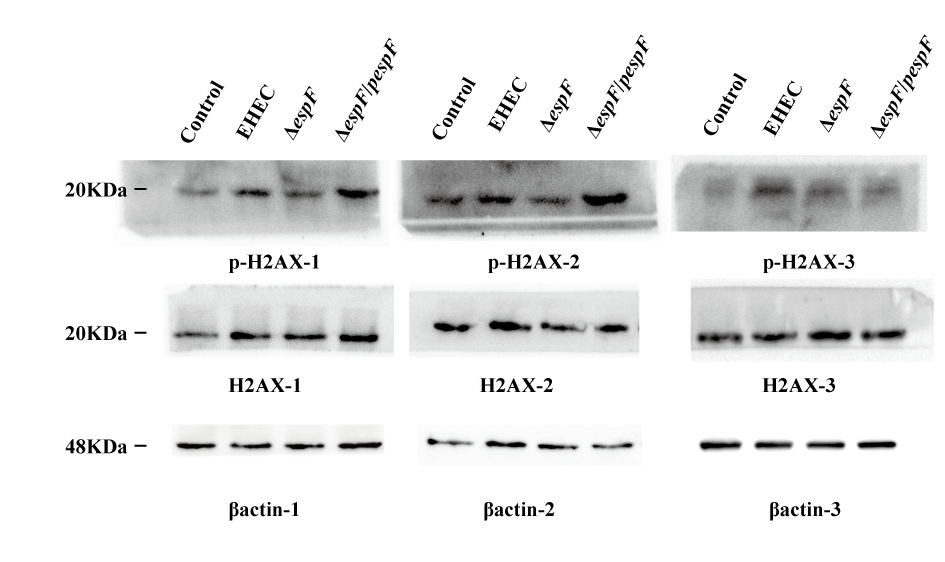

Supplement: Supplementary Figure 1 — Supplementary data for Figure 1. Three independent Western blots to detect p-H2AX and H2AX protein in Caco2 cells. [file Image_1.TIF]

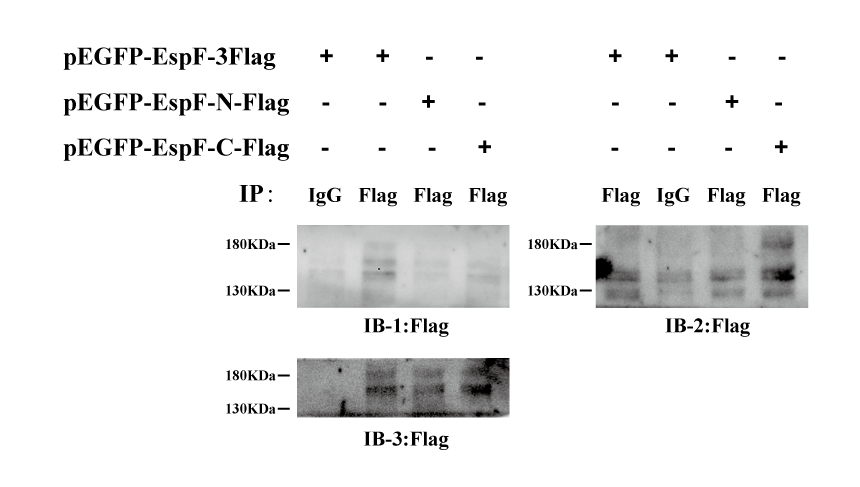

Supplement: Supplementary Figure 2 — Supplementary data for Figure 4. Three independent CoIP experiments to confirm the interaction between EspF, EspF-N, and the EspF-C terminus and SMC1 protein. [file Image_2.TIF]

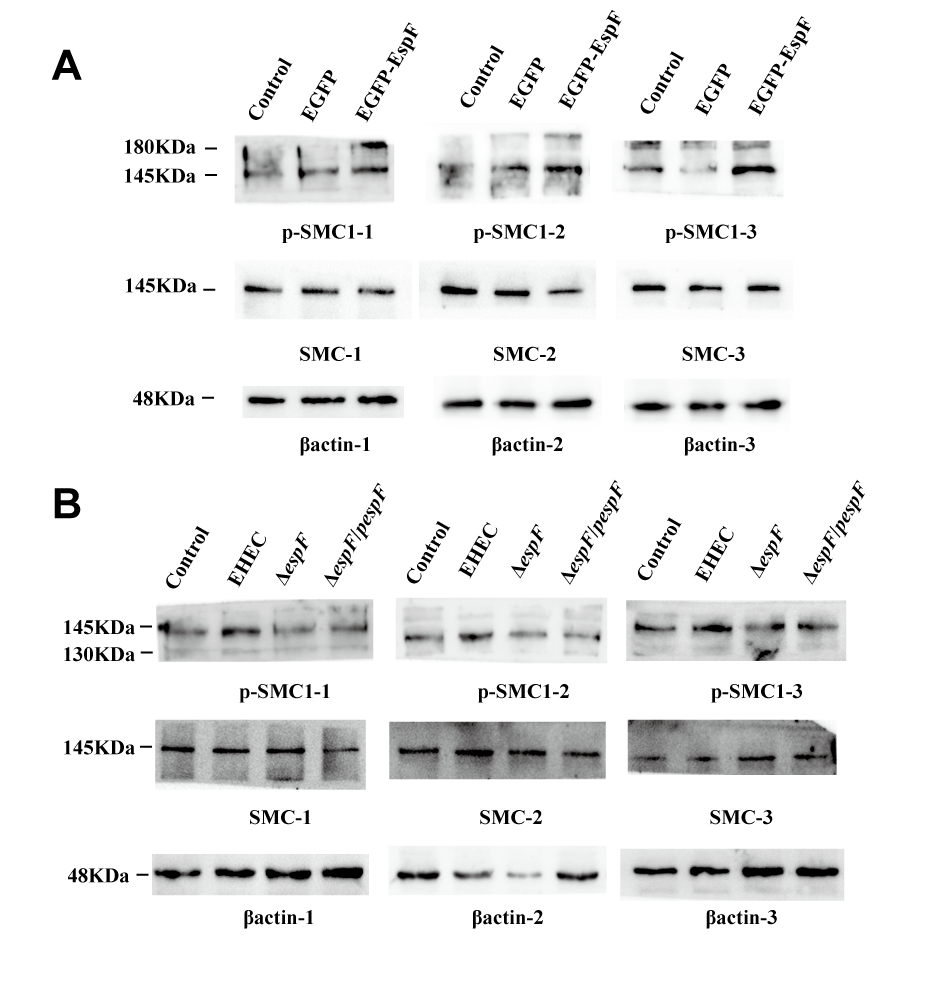

Supplement: Supplementary Figure 3 — Supplementary data for Figure 5. Three independent Western blots to detect p-SMC1 and SMC1 protein in Caco2 cells and HT-29 cells. [file Image_3.TIF]

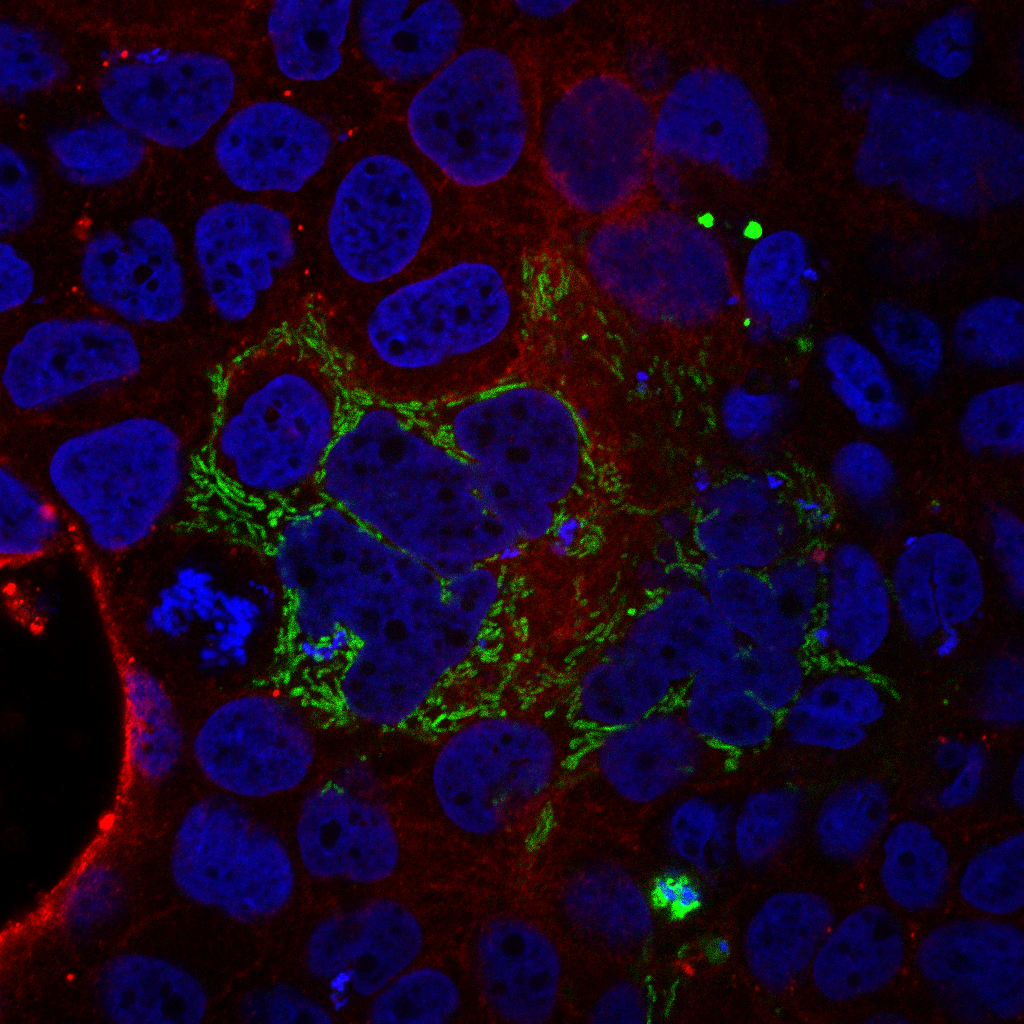

Supplement: Supplementary file 4 [file Image_4.TIF]

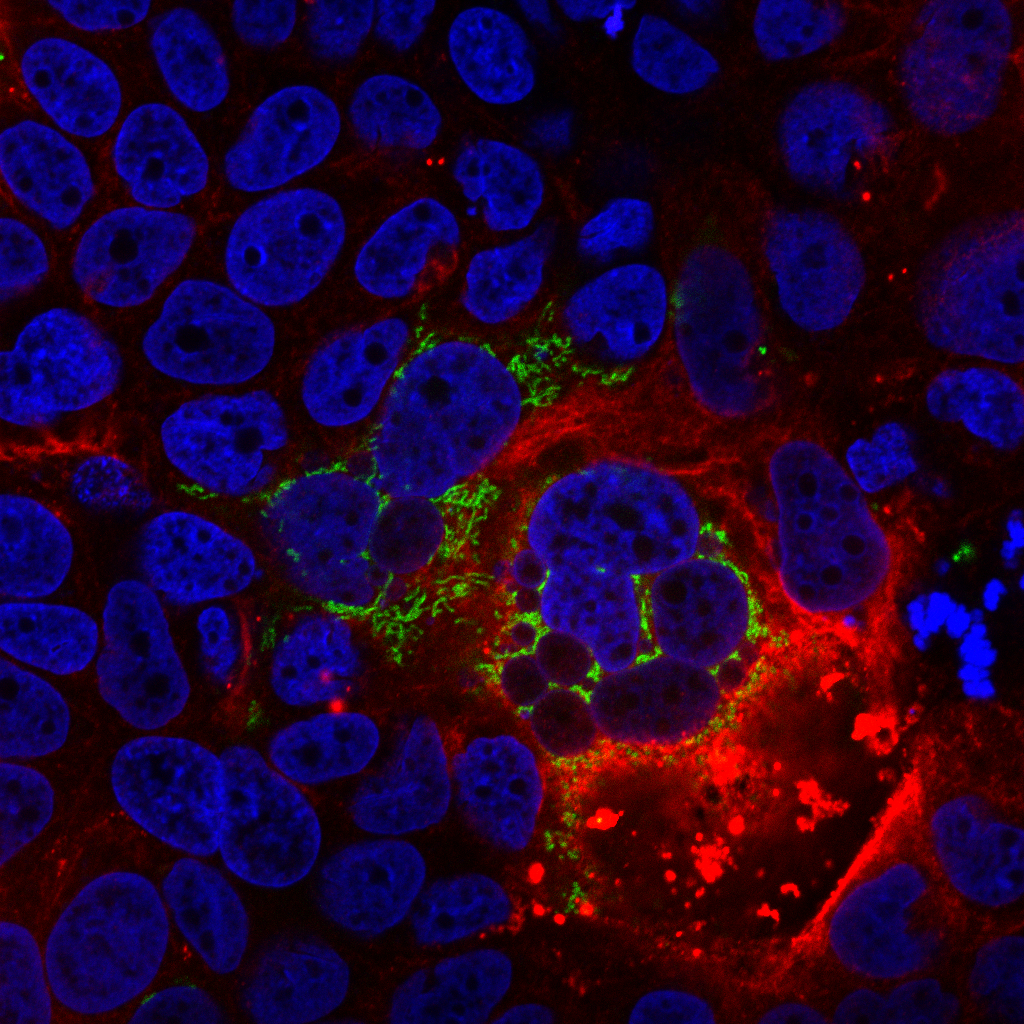

Supplement: Supplementary file 5 [file Image_5.TIF]

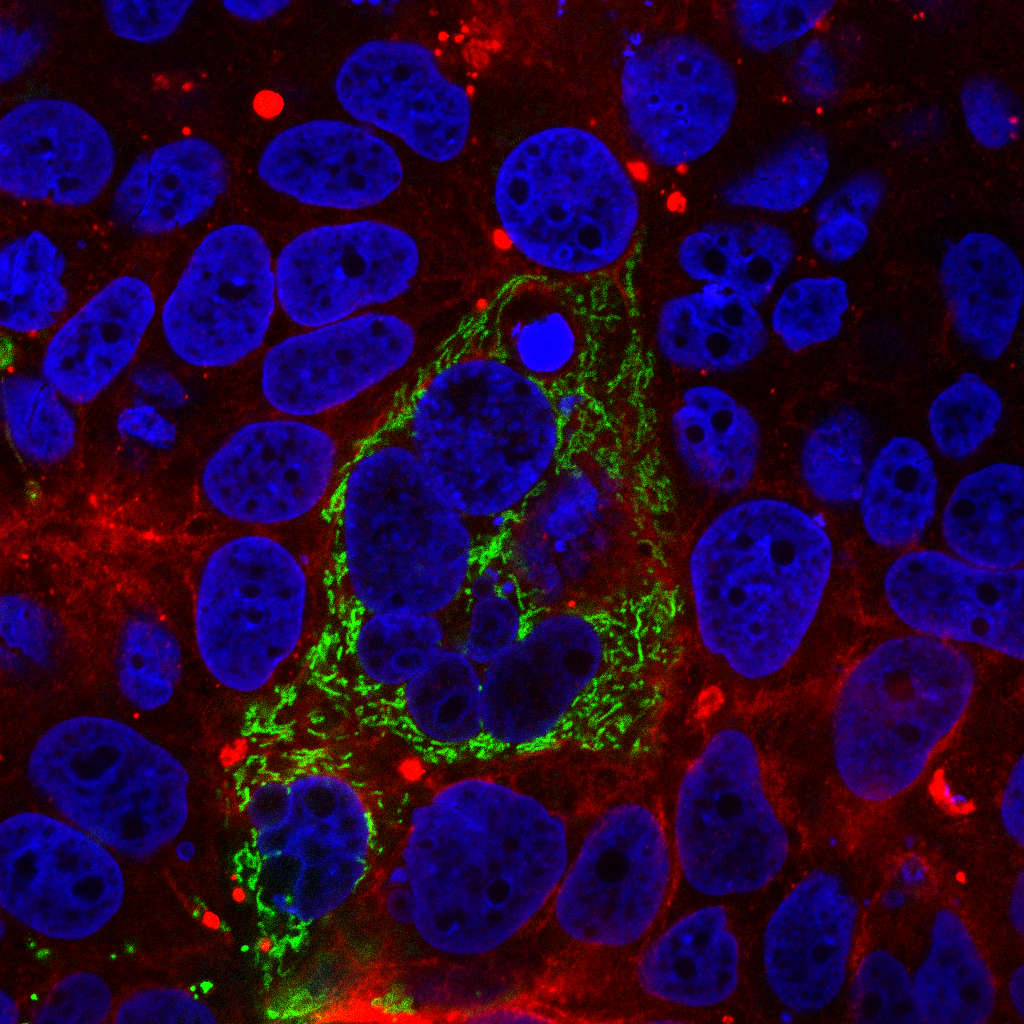

Supplement: Supplementary Figures 4–6 — Immunofluorescence figures of internalization phenomenon in Caco2 cells expressing EspF. [file Image_6.TIF]

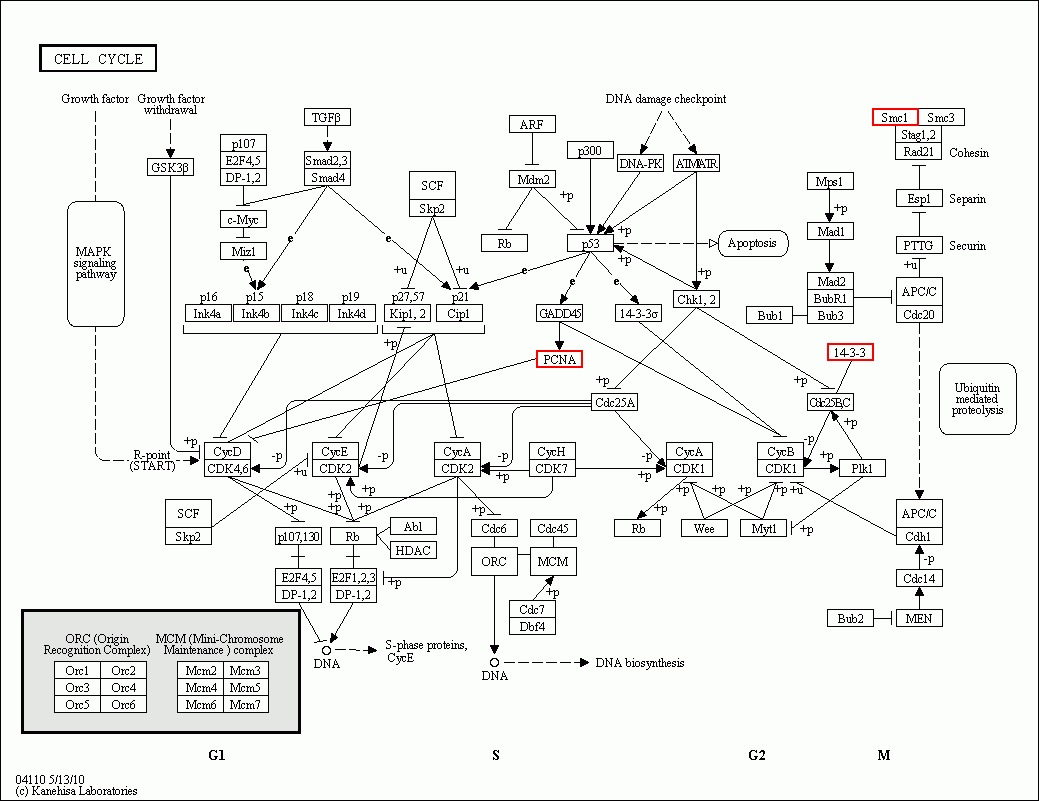

Supplement: Supplementary Figure 7 — iTRAQ analysis of the differential proteins of EHEC and ΔespF strains infected with HT29 cells in the cell cycle pathway. [file Image_7.JPEG]
